# Roscovitine effectively enhances antitumor activity of temozolomide *in vitro* and *in vivo* mediated by increased autophagy and Caspase-3 dependent apoptosis

**DOI:** 10.1038/s41598-019-41380-1

**Published:** 2019-03-21

**Authors:** Vimal Pandey, Nikhil Ranjan, Parimala Narne, Phanithi Prakash Babu

**Affiliations:** Laboratory of Neuroscience, Department of Biotechnology & Bioinformatics, School of Life Sciences, University of Hyderabad, Hyderabad, AP India

## Abstract

Gliomas are incurable solid tumors with extremely high relapse rate and definite mortality. As gliomas readily acquire resistance to only approved drug, temozolomide (TMZ), there is increasing need to overcome drug resistance by novel therapeutics or by repurposing the existing therapy. In the current study, we investigated antitumor efficacy of roscovitine, a Cdk inhibitor, in combination with TMZ *in vitro* (U87, U373, LN 18 and C6 cell lines) and *in vivo* (orthotopic glioma model in Wistar rats) glioma models. We observed that TMZ treatment following a pre-treatment with RSV significantly enhanced chemo-sensitivity and suppressed the growth of glioma cells by reducing Cdk-5 activity and simultaneous induction of autophagy and Caspase-3 mediated apoptosis. Additionally, reduced expression of Ki67, GFAP and markers of angiogenesis (CD31, VEGF) was observed in case of TMZ + RSV treatments. Also, presence of reactive astrocytes in peri-tumoral areas and in areas around blood vessels was completely diminished in TMZ + RSV treated brain sections. Taken together, results in the current study provide evidence that RSV in conjunction with TMZ restricts glioma growth, reduces angiogenesis and also eliminates reactive astrocytes thereby preventing the spread of glioma to adjacent healthy brain tissues and thus might be more potent therapeutic option for glioma.

## Introduction

Glioblastomas (GBM) comprise majority of malignant central nervous system tumors, with an annual incidence of 3.19 per 100,000 in the United States and a post-diagnosis 5-year survival rate of less than 5%^1^. It remains one of the most aggressive solid tumors and is highly resistant to conventional chemotherapy incurring a high relapse rate with a meager mean life expectancy of less than 14 months in afflicted individuals^2^. Despite the standard of care regimen comprising surgery, radiotherapy, and chemotherapy providing a successful initial treatment, disease recurrence is inevitable and almost always fatal in majority of GBM cases. Therefore, improved therapy for GBM either by novel therapeutics or by supplementing existing therapy is imperative. To reduce high drug development costs, researchers have largely become interested in repurposing already approved drugs. Some examples of drug-repurposing studies for GBM include ibudilast, metformin and chloroquine^3^.

Roscovitine (RSV), a cyclin-dependent kinase (Cdk) inhibitor, is a low molecular weight tri-substituted purine analogue which has been shown to inhibit Cdk 1, 2, 5, 7 and 9 at different concentrations^4–6^. It has been shown that RSV blocks the proliferation of various tumor cells, including that of neuronal cell types and tumor xenografts^7,8^. Several pre-clinical and clinical studies suggest that RSV is a well tolerated oral agent with therapeutic potential against a range of tumor types^7,9^. Low molecular weight of RSV facilitates its uptake, passage through blood brain barrier (BBB) and retention in brain^10^. It is apparently toxic to glioma cells while sparing normal astrocytes. It has been shown recently that sub-toxic concentrations of RSV can sensitize glioma cells that over-express the anti-apoptotic Bcl-2 or Bcl-xL to tumor-related apoptosis-inducing ligand^11^. Although, RSV monotherapy in cancer clinical trials have not been very encouraging, information regarding its synergistic cytotoxicity with several anticancer agents in multiple cancer types is substantial^7^. In line with this, RSV in combination with sapacitabine is currently undergoing clinical trials in advanced solid tumors (clinicaltrials.gov.in; NCT00999401).

Several studies have shown that among other Cdks, RSV is a potential inhibitor of Cdk5, the activity of which is indispensable for brain development^12^. Cdk5 plays a central role during synaptogenesis and neuro-transmission under physiological conditions^13–16^. However, excessive Cdk5 activation can result in neuronal dysfunction and death by varied mechanisms leading to neurodegeneration^17,18^. Increasing evidence substantiates the contribution of Cdk5 over expression in initiation of the DNA-damage response and DNA repair^19^. In many cancers Cdk5 inhibition or Cdk5 knockdown is shown to increase cytotoxicity and restore chemotherapeutic sensitivity^20–22^. Importantly, work by several groups suggests that Cdk5 correlates positively with glioma grades in human samples^23,24^. Thus, it becomes exciting to hypothesize that Cdk5 inhibition may be a valid strategy to bypass the resistance to chemotherapy and radiation therapy in glioma.

Though a significant amount of information exists regarding antitumor efficacy and synergism of RSV with numerous anticancer agents, reports investigating the effect of RSV in glioma are scarce^25^. Therefore, we investigated the effect of RSV alone and in combination with TMZ *in vitro* and *in vivo* glioma settings. We observed that RSV per se exerted significant anti-proliferative effect on glioma cell growth and RSV pretreatment sensitized the glioma cells to cytotoxic effects of TMZ. Additionally, RSV also reduced the number of reactive astrocytes and their localization around blood vessels significantly thereby restricting the spread of glioma cells to the healthy parts of brain. Also, combination therapy of TMZ + RSV reduced the expression of angiogenic markers CD31 and vascular endothelial growth factor (VEGF) *in vivo*. Interestingly, we observed that the anti-proliferative effect of TMZ + RSV on glioma was partly mediated by Cdk5 inhibition induced autophagy in conjunction with Caspase-3 mediated apoptosis, as evidenced by an increased expression of autophagy and apoptosis markers and increased survival upon pretreatment with 3-methyladenine (3-MA, early stage autophagy inhibitor) and Z-DEVD FMK, a Caspase-3 inhibitor.

## Results

### Roscovitine inhibits proliferation and potentiates the cytotoxic effects of TMZ on glioma cells *in vitro*

**Figure 1:**
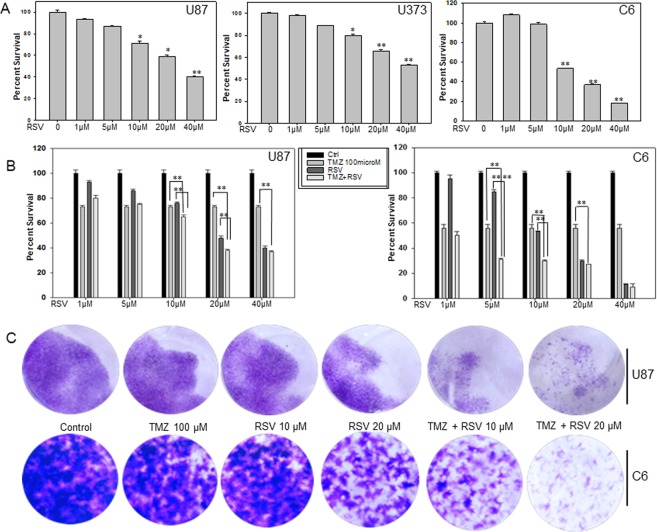
Roscovitine reduces glioma cell viability and potentiates the cytotoxic effects of TMZ on glioma cells *in vitro*. (**A**) Effect of RSV on cell viability in U87, U373 human glioma cell lines and C6, rat glioma cell line. The cells were treated with increasing concentrations of RSV for 72 h and cell viability was assessed by MTT assay. (**B**) Effect of TMZ and RSV co treatment on cell viability of U87 and C6 glioma cell lines. Cells were treated first with indicated concentrations of RSV followed by co-treatment with 100 µM TMZ. After 72 h incubation with the drugs cell viability was measured by MTT assay. (**C**) Long term colony formation assay for U87 and C6 cells after treatment with either TMZ, RSV or TMZ + RSV. The cells were treated with indicated concentrations of drug(s) for 72 h. After that medium was replaced with fresh DMEM supplemented with 10% FBS and cells were allowed to grow for 14 days with media change every third day. On 14^th^ day, colonies were fixed and visualized by staining with 0.05% crystal violet for 30 min. *p < 0.05, **p < 0.005.

Anti-proliferative activity of RSV has been confirmed in several tumor cell lines but reports regarding glioma are scarce. There is a single study reporting growth inhibition upon RSV treatment in several glioma cell lines. To confirm the effect of RSV on glioma cell lines we treated U87, U373 and LN18, human glioma cell lines and C6, a rat glioma cell line with increasing concentrations (1 µM to 40 µM) of RSV. We observed a dose-dependent decrease in percent survival of all the three cell lines tested. U87 cells showed a significant dose-dependent decrease in percent survival from approximately 93% to 40% whereas in U373 cells the decrease was from 98% to 53% with an increasing dose from 1 µM to 40 µM (Fig. 1A). C6 cells showed a decrease from 100% to 18% in response to treatment with increasing dose of RSV from 1 to 40 µM (Fig. 1A). The decreased percent survival was statistically significant at 10 µM, 20 µM and 40 µM RSV (p < 0.005) concentrations. The decreased percent survival was statistically significant at 10 µM, 20 µM (p < 0.05) and 40 µM (p < 0.005) concentrations of RSV in U87 cells. Also in U373 cells, the decrease in cell viability was statistically significant at 10 µM (p < 0.05), 20 µM and 40 µM (p < 0.005) RSV concentrations. Similar results were also observed in LN 18 glioma cells (Supplementary Fig. 1A). We further investigated the effect of RSV in combination with TMZ in these cells by MTT assay and long term colony formation assay. For these experiments we used TMZ concentration of 100 µM which represents the clinically relevant concentration of the drug^26^. We treated U87 and C6 cells with different concentration of RSV ranging from 0 to 40 µM followed by treatment with 100 µM TMZ. In U87 cells when combined drug treatment was given, we observed statistically significant reduction in percent survival in cells treated with 10 µM, 20 µM and 40 µM RSV (p < 0.005) as compared to TMZ alone treated cells (Fig. 1B). As can be seen in Fig. 1B, reduction in cell viability was not statistically significant at all the concentrations tested. When compared to TMZ-alone treated cells, in C6 cells, significant reduction in percent survival with combination therapy was observed at concentrations of 5 µM, 10 µM, 20 µM and 40 µM (p < 0.005) of RSV. When compared to RSV-alone treated cells, potentiation of cytotoxicity in combination therapy was significant at 5 and 10 µM (p < 0.005) RSV concentrations in both the U87 and C6 cell lines. Potentiation of TMZ cytotoxicity with RSV concentration was also observed in LN18 cells at similar concentrations (Supplementary Fig. 1B). Based on isobologram analysis and combination index (CI) values, the enhanced cytotoxicity at TMZ concentration of 100 µM appears to be synergistic with a CI value of 0.7 in U87 cells (Supplementary Fig. 1C). These observations from MTT assay were complemented by long term cytotoxicity assay. As observed in Fig. 1C, significant reduction in the number of colonies was observed in cells treated with a combination of TMZ and RSV in both U87 and C6 cell lines (Fig. 1C).

The colony formation assay confirmed that while both TMZ and RSV alone at experimental concentrations inhibited glioma growth, combination of TMZ with RSV had better anti-proliferative capacity in the glioma cell lines tested.

### Roscovitine alone or in combination with TMZ decreases glioma progression *in vivo*

**Figure 2:**
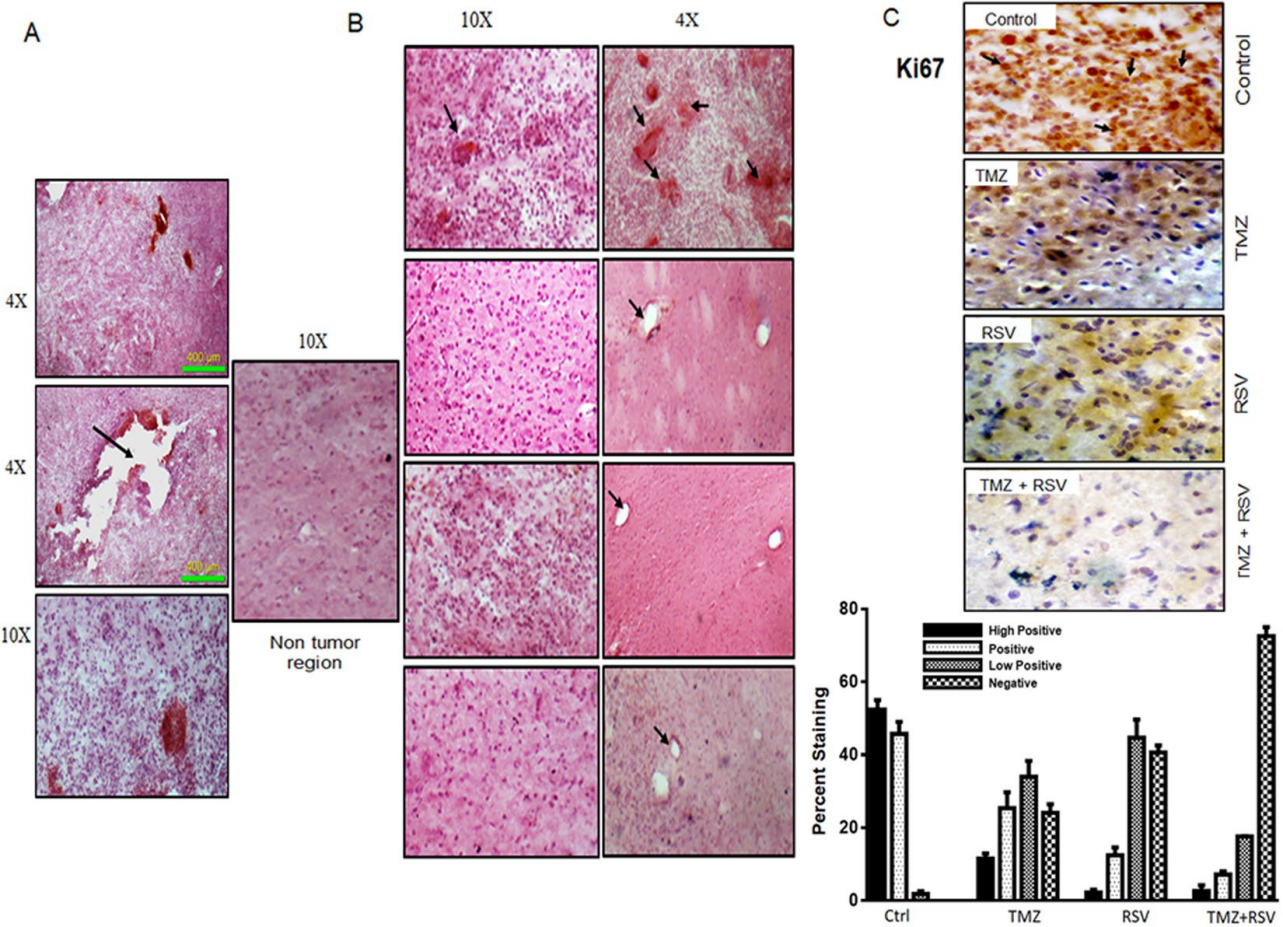
Roscovitine (RSV) alone or in combination with Temozolomide (TMZ) restricts glioma progression *in vivo*. (**A**) H and E staining of tumor sections on 7^th^ day after tumor implantation was done in 2 randomly selected rats to confirm the development of tumor. Sections are shown at different magnification to show the spread of tumor from the site of injection (black arrow). (**B**) H and E staining of tumor sections (5–10 µ) from vehicle, TMZ, RSV and TMZ + RSV (from top to bottom) treated rats. After completion of the respective treatment cycles, rats were sacrificed. Brains were harvested and fixed in 4% PFA followed by sucrose gradient dehydration and cryosectioning. The sections thus obtained were used for H and E staining. Images were captured at 10 × and 4 × . Black arrows show the extensive presence of hemorrhages in vehicle treated tumors whereas hemorrhages were totally absent in all the treated tumors. (**C**) 25–30 µ sections were used for Immunohistochemical staining for Ki67 in tumor sections from vehicle, TMZ, RSV and TMZ + RSV treated rats. Black arrow shows clear nuclear staining for Ki67 in tumors from vehicle treated rats. Representative images of tumor sections are shown for each group.

Considering the significant anti-proliferative effect of RSV and TMZ + RSV *in vitro*, we tested its relevance *in vivo*. *In vivo* glioma model using C6 cell line was established in Wistar rats using stereotaxic apparatus. Development of tumor was confirmed by randomly selecting two rats and sacrificing them on 7^th^ day after implantation of tumor. Brains of these rats were dissected, processed and subjected to H and E staining and observed under the microscope to confirm the presence of tumor (Fig. 2A).

Tumor bearing rats were divided into four groups and were vehicle untreated (Control) or treated with TMZ, RSV or a combination of both the drugs (RSV + TMZ). TMZ was administered at a dose of 10 mg/kg/day and RSV was given 30 min prior to TMZ at a dose of 25 mg/Kg/day (on alternate days in 3 doses). Rats were then sacrificed on 8^th^ day from the start of the treatment (14^th^ day of tumor implantation) and brains were dissected and stored in 4% PFA followed by dehydration in sucrose gradients. Further, these brains were sectioned using cryo-microtome and sections were subjected to H and E staining or IHC staining. Histological analysis with H and E staining of these sections showed significantly decreased cell numbers in tumor sections from TMZ, RSV and TMZ + RSV treated rats when compared to the control vehicle treated rats. However, as can be visualized in Fig. 2A, maximum inhibition of tumor proliferation was observed in TMZ + RSV treated group. In fact, the cell density in tumor sections from this group was almost similar to non-tumor region. H and E stained tumor sections in the vehicle-treated rats showed presence of severe haemorrhages in blood vessels which were not observed in any of the treated groups (TMZ, RSV and TMZ + RSV) (Fig. 2B). Immunohistochemical staining for Ki67, which is a known marker of active proliferation in cancer cells including glioma cells, also confirmed a significantly reduced progressive potential of C6 cells in tumor sections from treated rats (TMZ, RSV and TMZ + RSV) as compared to vehicle-treated rats (Control). Clear significant nuclear positivity for Ki67 was observed in tumor sections from vehicle-treated rats whereas negligible nuclear staining for Ki67 was observed in tumor sections of all the three treated groups (Fig. 2C and Supplementary Table 1).

### RSV treatment alone or in combination with TMZ decreases reactive astrocytes and reduces angiogenesis

**Figure 3:**
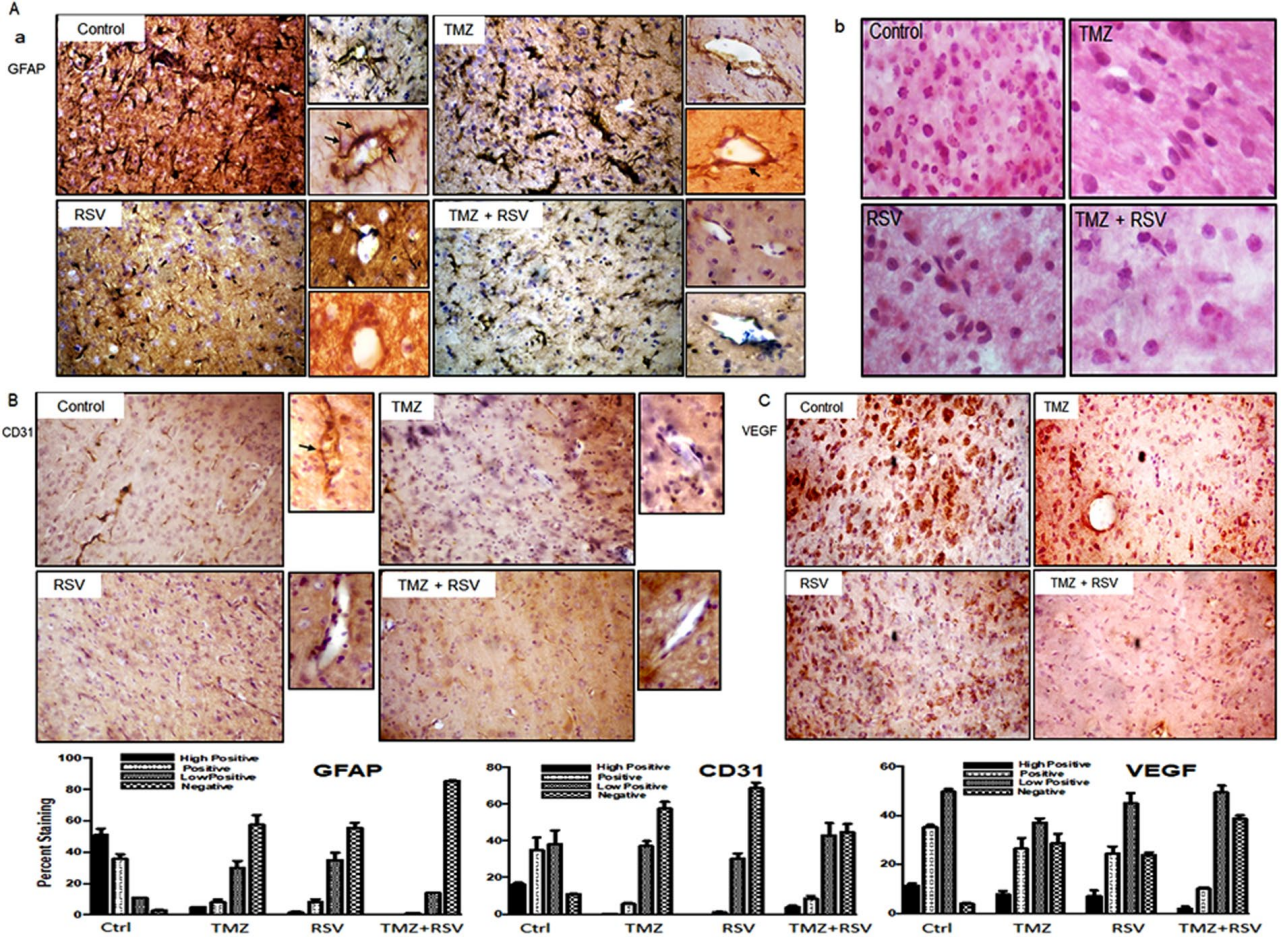
Roscovitine (RSV) treatment effectively reduces the density of reactive astrocytes and markers of angiogenesis. (**A**) Immunohistochemical staining for GFAP in tumor sections from vehicle, TMZ, RSV and TMZ + RSV treated rats. Black arrows depict the reactive astrocytes with long and short processes stained intensly for GFAP. Appended images show the prominence of reactive astrocytes around blood vessels for the respective groups. (**B**) H and E staining of tumor sections from vehicle, TMZ, RSV and TMZ + RSV treated rats at 40X magnification. (**C**) Nuclear morphology typical of reactive astrocytes are abundant in vehicle treated tumor sections. (**C**) Immunohistochemical staining for CD31 (**A**) and VEGF (**B**) in tumor sections from vehicle, TMZ, RSV and TMZ + RSV treated rats. Appending the CD31 IHC images are magnified images showing strong reactivity of CD31 (black arrow) around blood vessels in Vehicle treated sections.

GFAP is a marker for glial cells. Immunohistochemical staining with GFAP usually shows cytoplasmic staining in glial sections. However, according to literature, GFAP staining can also be used as a marker for reactive astrocytes, as the reactive astrocytes stain intensely positive for GFAP and their long and short processes also stain positive for GFAP^27^. Interestingly, the sections of tumors from control untreated rats showed overwhelming abundance of reactive astrocytes in peritumoral areas (Fig. 3Aa, Supplementary Table 2). This observation is also supported by gemistocytic nuclear morphology in these sections which is specific for reactive astrocytes (Fig. 3Ab). Reactive astrocytes were also conspicuous around the blood vessels in tumor sections from vehicle treated rats (Fig. 3Aa, Black arrows). Reactive astrocytes potentiate tumor aggressiveness, possibly by augmenting inflammatory responses crucial to the progression and spread of tumor. The number of reactive astrocytes was relatively reduced in TMZ treated tumor sections but surprisingly, in RSV treated sections, they were negligible. Similarly, in tumor sections which received combination therapy of TMZ and RSV, the reactive astrocytes were totally reduced in size and numbers (Fig. 3Aa).

Angiogenesis is an important survival strategy of tumors that enables them to obtain the supply of nutrients directly from the blood. GBM is classified as a highly vascularised tumor. To check whether current treatment regimes had any effect on angiogenesis in gliomas, we checked for the expression of CD31 and VEGF, which are important angiogenic markers for glioma. We observed a significant decrease in the expression level of CD31 in TMZ, RSV and TMZ + RSV treated tumor sections when compared to control tumor sections (Fig. 3B, Supplementary Table 3). Also, in control tumors, prominent staining of CD31 around the blood vessels was observed (Black arrows, Fig. 3B). However, expression pattern of VEGF in these tumors was observed to be slightly variable. Although TMZ and RSV treated tumor sections showed positive VEGF staining, the intensity of staining in TMZ-treated and RSV-treated tumor sections was much reduced when compared to vehicle-treated control tumor sections. In TMZ + RSV treated tumor sections, very weak VEGF staining was observed (Fig. 3C, Supplementary Table 4).

### Cdk5 inhibition decreases glioma cell survival and induces autophagy

**Figure 4:**
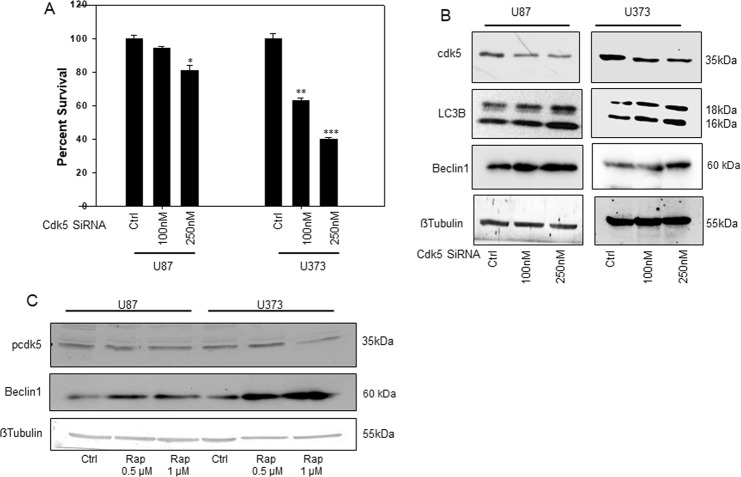
Cdk5 knockdown reduces glioma cell viability and induces autophagy. (**A**) Effect of Cdk5 knockdown on percent survival of U87 and U373 cell lines using specific SiRNA. Cells were treated with indicated concentrations of Cdk5 specific SiRNA and control SiRNA. Cell viability was assessed by MTT assay 48 h post transfection. (**B**) Immuno-blots depicting the knockdown of Cdk5 protein and increased expression of LC3B upon Cdk5 knockdown in U87 and U373 cells. Whole cell lysates prepared from U87 and U373 cells, 48 h post transfection were immune blotted for the expression levels of Cdk5, LC3B and beta actin. (**C**) Immuno blot depicting changes in the expression of pCdk5 upon treatment with Rapamycin (Rap) in U87 and U373 cells. *p < 0.05, **p < 0.001, ***p < 0.0001.

Existing literature demonstrates that Cdk5 is highly up-regulated in gliomas when compared to normal human astrocytes. Also, as described earlier, RSV is known to effectively inhibit Cdk5 activity^28^. To investigate the role of Cdk5 expression and activity in glioma cells, SiRNA mediated knockdown approach was used. Interestingly, SiRNA-mediated knockdown of Cdk5 resulted in a notable dose-dependent decrease in percent survival in U87 and U373 cells. A significant decrease of approximately 60% (p < 0.001) in percent survival of U373 cells was observed whereas in U87 percent survival decreased by 25% (p < 0.05). Thus, it was observed that Cdk5 expression is crucial to the survival of these glioma cell lines and down regulation of Cdk5 levels by specific SiRNA reduces the proliferation of U87 and U373 cells (Fig. 4A). Reports suggest a possible role of Cdk5 in autophagy regulation in neurons^29^. We therefore investigated the possible role of Cdk5 in autophagy regulation in glioma cell lines. For this, we checked the expression level of LC3B and Beclin 1 after SiRNA mediated knockdown of Cdk5 in U87 and U373 cells. Interestingly, we observed that knockdown of Cdk5 was associated with significantly increased expression of both LC3B and Beclin 1, well known autophagy markers (Fig. 4B, Supplementary Fig. 2). Thus, similar to neuronal cells, there appears to be a direct autophagy inhibitory function of Cdk5 in glioma cells. To check whether Cdk5 regulates autophagy and not vice versa, we induced autophagy in U87 and U373 cells by treatment with rapamycin, which is an established inducer of autophagy. Following treatment with rapamycin, we checked phospho-Cdk5 (pCdk5) levels in U87 and U373 cells treated with rapamycin. Phosphorylation levels of Cdk5 are an indirect measure of its kinase activity. Interestingly, there was a reduced Cdk5 activity in rapamycin treated cells as evidenced from decreased expression of pCdk5 in rapamycin treated cells. 1 µM concentration of rapamycin in both the cell lines inhibited pCdk5 levels but decrease was more marked in U373 cells which shows that U87 cells are more resistant to induction of rapamycin induced autophagy (Fig. 4C, Supplementary Fig. 2). Immunoblotting for Beclin 1 indeed showed a higher increase in the protein expression levels in U373 cells when compared to U87 cells (Fig. 4C, Supplementary Fig. 2). Based on these results, it appears that autophagy and Cdk5 activity are co-ordinately regulated by each other and Cdk5 has a complex role in autophagy regulation in glioma cells.

### Temozolomide treatment down regulates Cdk5 activity and induces autophagy in glioma cells *in vitro*

**Figure 5:**
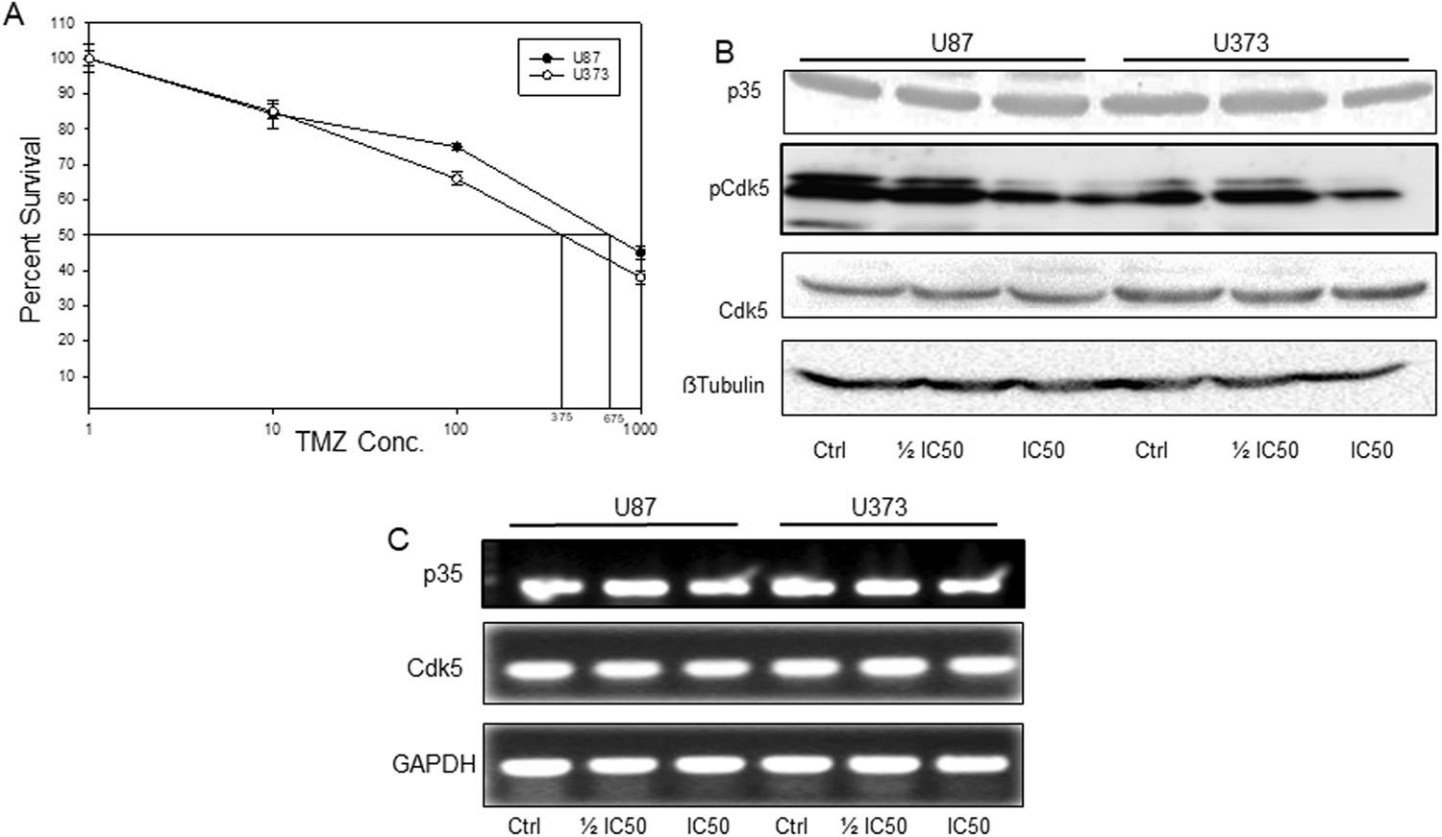
Temozolomide (TMZ) down regulates Cdk5 activity and induces autophagy in glioma cells. (**A**) IC 50 values of TMZ for U87 and U373 cell lines. U87 and U373 cells were treated with increasing concentrations of TMZ for 72 h followed by MTT assay to determine percent cell survival. The concentrations corresponding to 50% cell death in each cell line was determined from the graph and taken as IC 50 values corresponding to the cell line. (**B**) Immuno-blots showing the effect of TMZ IC50 and $1/2$ IC50 on the expression levels of p35 and pCdk5 in U87 and U373 cells. Cells were treated with indicated concentration of TMZ for 72 h, whole cell lysates were used for immunoblotting with indicated antibodies. (**C**) Semi quantitative PCR for p35 and Cdk5 in U87 and U373 cells in control and TMZ treated conditions.

TMZ is the only chemotherapeutic drug currently in use for glioma patients. TMZ, a DNA damaging agent, is known to induce apoptosis and autophagy in glioma cells. Increasing evidence substantiates the contribution of Cdk5 in initiation of the DNA-damage response (DDR), DNA repair and autophagy. Thus, we explored the effect of TMZ treatment on Cdk5 activity and autophagy in glioma cells *in vitro*. For this we first estimated the IC50 drug concentration of TMZ in U87 and U373 cells by MTT assay. We observed that U87 cells were more resistant to TMZ treatment with an IC50 value of 650 µM and U373 cells were sensitive with an IC50 value of 375 µM (Fig. 5A). At respective $1/2$ IC50 and IC50 concentrations, we observed a dose dependent decreased expression of pCdk5 in both the cell lines (Fig. 5B). In addition, we checked the expression levels of p35, an effector protein of Cdk5 under these conditions. We observed a down regulation of p35 expression upon treatment with different TMZ concentrations. However, the decrease in p35 expression was not dose-dependent (Fig. 5B). Following this observation we checked the mRNA expression of p35 and Cdk5 in U87 and U373 cells treated with indicated concentrations of the drug. However, we did not find any significant changes at mRNA levels upon treatment with TMZ (Fig. 5C). Thus, it appears that treatment with TMZ does not alter transcriptional levels of Cdk5 but mainly affects the activity of Cdk5 by modulating the activating mechanisms like phosphorylation of Cdk5.

**Figure 6:**
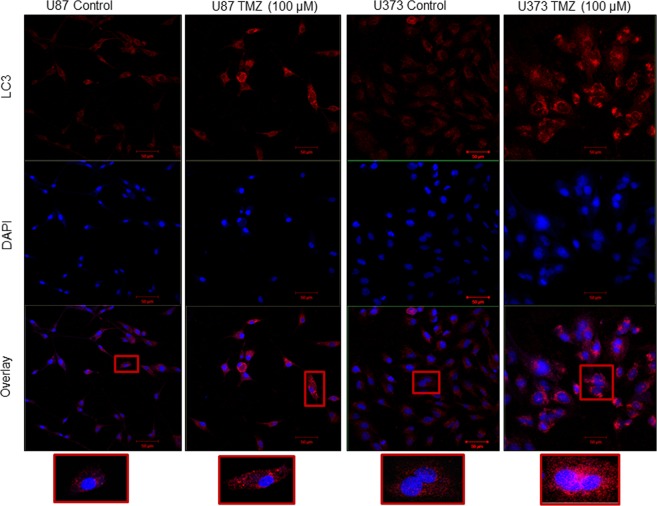
Immuno-fluorescent staining for LC3B in U87 and U373 untreated and TMZ treated cells. Cells were treated with IC50 values of TMZ for 72 h. Following this cells were fixed and processed for immunofluorescence of LC3B protein. Bars represent 50 µm.

To validate autophagy induction in response to TMZ in our model system, we checked for the LC3B expression by immunofluorescence. In line with the existing literature, as depicted clearly in single cell images and quantification data, we observed that U87 and U373 cells treated with TMZ showed an increase in LC3 staining compared to control cells (Fig. 6, Supplementary Fig. 3).

### Enhanced cytotoxicity of TMZ + RSV *in vitro* and *in vivo* is mediated by increased autophagy and apoptosis

**Figure 7:**
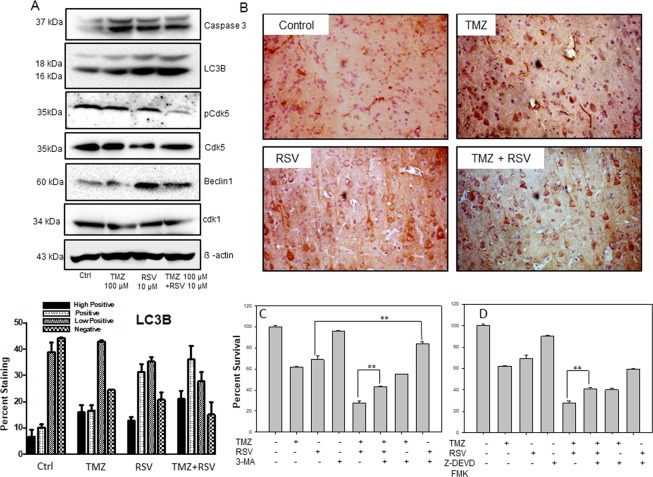
Enhanced cytotoxicity of TMZ + RSV *in vitro* and *in vivo* is mediated by increased autophagy. (**A**) Immunoblotting of apoptotic and autophagic marker proteins in control and drug treated C6 cells. Cells were treated with indicated drug(s) concentrations for 72 h and whole cell lysates were prepared. Protein expression levels of Caspase 3, LC3B, Beclin1, pCdk5 and beta actin were assessed by immunoblotting. (**B**) Immunohistochemical staining for autophagy marker, LC3B in tumor sections from vehicle, TMZ, RSV and TMZ + RSV treated rats. (**C**) Effect of 3-MA, an early stage autophagy inhibitor on the cytotoxicity of various drug treatments as indicated in the figure. (**D**) Effect of Caspase 3 inhibitor (Z-DEVD FMK) on the cytotoxicity of various drug treatments as indicated in the figure. For C and D, C6 cells were pretreated with indicated concentrations of inhibitors for 2 h and then treated with TMZ, RSV or TMZ + RSV for 72 h. This was followed MTT assay to determine percent cell survival. *p < 0.05, **p < 0.005.

As described in *in vitro* results, TMZ in glioma cells induces autophagy. Further, Cdk5 inhibition in glioma cells also leads to increased autophagy. Autophagy has been proposed as one of the cell death mechanism in several cases. Thus it is plausible that a cumulative increase in autophagy might be involved in increased cytotoxicity of TMZ when used in combination with RSV. Thus, we checked the expression of apoptosis and autophagy-related markers in C6 cells under control, TMZ, RSV and TMZ + RSV treatment conditions. First, we checked the status of Cdk5 activation in response to these drug treatments. We observed a reduced expression of pCdk5 in TMZ and RSV treated cells when compared to control cells. However, as can be observed, maximum inhibition of Cdk5 activity was observed in case of combination treatment with TMZ + RSV (Fig. 7A). Cdk1 is another cyclin dependent kinase which is known to be affected by RSV treatment. The protein expression levels of Cdk1 were reduced in RSV treated cells. However, the decrease in TMZ and TMZ + RSV treated cells was not significant. We observed an increased Caspase 3 cleavage in TMZ treated cells when compared to control cells. Caspase 3 cleavage was also observed in RSV and TMZ + RSV treated cells. Importantly, we observed a progressive increase in LC3B expression and cleavage in TMZ, RSV and TMZ + RSV treated cells. We also observed an increased expression of Beclin1, a known positive regulator of autophagy in RSV and TMZ + RSV treated cells (Fig. 7A). To validate the *in vivo* relevance of these findings we performed IHC staining for LC3B in the tumor sections from vehicle treated and drug (s) treated rats. We observed increased autophagy as inferred from IHC staining for LC3B in the treated groups as compared to untreated section. TMZ-treated tumor sections showed moderate positivity for LC3B whereas RSV and TMZ + RSV treated tumor sections showed a signficant increase in LC3B staining when compared to vehicle treated tumor sections (Fig. 7B, Supplementary Table 5). To further confirm the involvement of autophagy in TMZ + RSV mediated cell death, we treated C6 glioma cells with various autophagy inhibitors. For this C6 cells were first pre-treated with either 3MA (1 mM), BafA1 (10 nM) or CQ (20 µM) for 2 h followed by treatment with either TMZ (100 µM), RSV (20 µM) or TMZ + RSV for 72 h. We observed that treatment with 3MA, which inhibits autophagy at early stages, was able to partially rescue C6 cells from RSV and TMZ + RSV mediated cell death. There was an increase in percent survival in 3MA pre-treated + TMZ + RSV treated cells (43%) when compared to only TMZ + RSV treated cells (28%) (Fig. 7C). Also, 3MA treatment reduced the percent cell death in RSV treated cells. Surprisingly, 3MA pre-treatment was not able to rescue cells in only TMZ treated cells. Also, pre-treatment with late stage autophagy inhibitor BafA1 or CQ was unable to revert the cell death. In fact, there was an enhanced cell death in BafA1 or CQ pre-treated cells (Supplementary Fig. 4A and B). As we observed only partial reversal of cell death by treatment with 3MA and also based on the observation that there is an increase in Caspase-3 cleavage in all the treated conditions, we also checked the effect of Caspase-3 inhibitor and total Caspase inhibitors on the percent survival of C6 cells treated with TMZ, RSV or TMZ + RSV. Not surprising, we again observed a partial reversal of cell death in TMZ + RSV treated cells which were pre-treated with Caspase-3 inhibitor (Fig. 7D). However, this was not observed in cells pre-treated with total Caspase inhibitor (Supplementary Fig. 4C). Thus, increased cell death in combination therapy of TMZ + RSV could be attributed to a combined effect of increased autophagy and Caspase-3 dependent apoptosis in these cells as evident from *in vitro* and *in vivo* data described.

## Discussion

TMZ is the only chemotherapeutic drug currently in use for glioma patients. However, drug resistance is a major challenge for patients undergoing chemotherapy with TMZ. From promising preclinical evidence, several monotherapy alternatives to treat these tumors have been evaluated in clinical trials. However, these single targeted therapies have not shown great promise in human trials due to acquired resistance of gliomas to these monotherapies. In such scenario, synergistic multidrug targeting might allow better therapeutic outcomes^30^. In the current study, we tested the aforementioned hypothesis using RSV in combination therapy with TMZ for glioma *in vitro* and *in vivo*. RSV was selected in the current study based on following considerations (1) Several preclinical and clinical studies suggest that RSV is well tolerated oral agent with therapeutic potential against a range of tumor types^7,9^. (2) Owing to its low molecular weight and structural similarity with purines, it can readily cross blood brain barrier and is efficiently retained in brain at concentrations which are cytotoxic to glioma cells^10^. (3) Additionally, RSV has demonstrated synergistic effects on the cytotoxicity of several anti-cancer agents by variable mechanisms^7^.

In the current study, we observed that upon treatment with increasing doses of RSV, significant dose-dependent inhibition of cell and tumor growth occurs *in vitro* and *in vivo* glioma models respectively. In fact, as a single agent, RSV at 40 µM concentration resulted in more than 50% inhibition of cell proliferation in these cell lines. However, previous studies have shown that sub toxic concentration of RSV for brain lies in the range of 5–20 µM^11^. Interestingly, we observed maximum potentiation of TMZ cytotoxicity at 10 and 20 µM RSV concentrations which lie within the sub-toxic concentration range. The sequential drug treatment regime followed in this study was based on already published literature advocating the maximum beneficial effect of RSV as a pretreatment^21^. Inhibition of cell growth in long term colony formation assay supplements our MTT data and majorly reveals that increased cytotoxicity of TMZ by RSV pretreatment is irreversible and persists even after withdrawal of drug. In keeping with *in vitro* findings, RSV potentiated the cytotoxic effects of TMZ *in vivo*. To start with, treatment with either TMZ or RSV were quite effective in inhibiting the proliferation of glioma tumor when compared to vehicle treated group. However, the inhibition of tumor growth was much lesser when compared to TMZ + RSV treated group of rats. In the rat glioma model, though TMZ was able to suppress the growth of tumor cells, complete inhibition was far from being reached at the dosage used. Similar was the case for RSV monotherapy. Nevertheless, combination therapy of TMZ + RSV was potentially effective in near-complete inhibition of tumor growth as evidenced by cellular density and morphology together with Ki67 IHC staining.

In an attempt to identify the possible mechanisms involved in this enhanced inhibition of tumor growth in TMZ + RSV treated rats, we checked for the presence of reactive astrocytes (an essential feature of aggressive gliomas) and angiogenesis. Reactive astrocytes create an inflammatory microenvironment in the areas surrounding the tumor and help in progression and spread of tumor by activating MMPs which degrade extracellular matrix and eventually help in the spread of tumor to adjacent healthy areas in case of brain tumors^27^. In accordance with previous reports, IHC with GFAP revealed a dense population of reactive astrocytes in the peri tumoral region and around the blood vessels in control tumor section^27,31^. Surprisingly, treatment with RSV and TMZ + RSV showed almost complete elimination of reactive astrocytes from peri-tumoral areas and blood vessels. In addition to reducing the population of reactive astrocytes, treatment with TMZ, RSV and TMZ + RSV also resulted in highly reduced angiogenesis as depicted by the IHC analysis of angiogenic markers, CD31 and VEGF in tumor sections. In response to the needs of nutrients and oxygen from the blood to grow and spread, angiogenesis is a process that occurs frequently in tumor regions. In the case of GBM, there is a high proliferative activity with infiltration into the surrounding tissues, since it is among the most highly vascularized tumors. Reports by Yushan *et al*.^23^, and Liu *et al*.^24^, show that Cdk5 is highly up-regulated in gliomas as compared to normal astrocytes and hence might be involved in the tumorigenic potential and rapid proliferation of glioma cells. However, there are no reports demonstrating the effects of Cdk5 knockdown in gliomas. Based on the aforementioned reports we observed that knockdown of Cdk5 effectively reduced the viability of glioma cells. Another observation (previously unreported) in the current study is that direct knockdown of Cdk5 induces autophagy in glioma cell lines. Beclin 1 expression levels showed that U87 cells were more resistant to autophagy induction in response to rapamycin and were also resistant to TMZ treatment as depicted by higher IC50 value. However, they were observed to be more responsive to TMZ + RSV combination therapy and showed synergistic decrease in percent survival. The reduced activity and expression levels of pCdk5 and p35 were not associated with changes in mRNA expression. Our data is supported by recent literature which has implicated Cdk5 in negative autophagy regulation by inhibiting the Class3PI3K pathway in neurodegenerative conditions^32^. Based on our findings we speculate that Cdk5 and autophagy are intricately connected in GBM cells and in fact coordinately regulate each other. Regarding this, we observed that autophagy induction by rapamycin leads to decreased expression of pCdk5 and an increase in Beclin 1 protein expression levels in glioma cells. Our data also demonstrates that reduced Cdk5 activity and altered downstream signaling may be one of the mechanisms mediating the cytotoxic effects of TMZ in glioma cells.

*In vitro* immunoblotting for pCdk5 level confirms the inhibition of Cdk5 activity in response to TMZ or RSV treatments. Immunoblotting for apoptosis and autophagy markers demonstrate a clearly increased autophagy induction in the cells treated with RSV and TMZ + RSV. In GBM, TMZ induced autophagy is putative. Recent studies indicate that autophagy-related signature can be used as an independent predictor of survival outcomes in glioma patients and could be potential therapeutic target in glioma^33,34^. Autophagy has basically been proposed as a cell survival mechanism under stress conditions. However, it has been shown that autophagy can perform seemingly opposite roles during tumorigenesis and chemotherapy as a pro-survival or a pro-death mechanism through mechanisms which are largely unknown^35^. With respect to autophagy induced by TMZ treatment, our results are largely in confluence with earlier studies which show that autophagy induced in TMZ monotherapy is largely cyto-protective^36^ Fig. 7C. On the contrary, it was observed that autophagy can easily trigger cell death in case of combination treatment^37,38^. With respect to the autophagy induced in case of RSV and TMZ + RSV combination treatment, our study undoubtedly supports the concept of autophagy mediated cell death. In line with this, *in vivo* immuno-staining of tumor sections with LC3B antibody, and increased survival in cells pretreated with 3MA confirmed our *in vitro* findings concerning autophagy as one of the mechanisms of cell death in glioma cells after treatment with RSV and TMZ + RSV combination therapy. However, in the current treatment regime autophagy is definitely not the sole cause of enhanced cytotoxicity for tumor cells. As can be inferred from partial increase in cell survival in case of Caspase-3 pretreatment followed by TMZ + RSV treatment, enhanced cytotoxicity in this case can be attributed to parallel increase in autophagy and apoptosis simultaneously. Recent literature shows that in addition to being an alternative mechanism to cell death when apoptosis is inhibited, autophagy in some cases can in fact, precede or act in parallel with apoptosis^39^. Similar case was observed by Ren *et al*., wherein FK-16, derived from the anticancer peptide LL-37, induced Caspase-independent apoptosis and autophagic cell death in colon cancer cells^40^.

To conclude, in the present study, for the first time, we pin down the growth-inhibitory impact of RSV and potentiation of TMZ cytotoxicity by RSV in *in vitro* and *in vivo* glioma models. We show that glioma growth inhibition can be attributed to simultaneous increase in autophagy and Caspase-3 dependent apoptosis, reduced angiogenesis and decreased number of reactive astrocytes thereby restricting the spread of GBM tumors to adjacent healthy tissue. We also provide preliminary evidence regarding coordinate regulation of autophagy and pCdk5 and reveal the direct involvement of pCdk5 in autophagy regulation and glioma cell death. Though further research is needed to understand mechanisms underlying Cdk5-mediated regulation of autophagy in glioma, our study definitely establishes this as a mechanism of RSV and TMZ + RSV mediated tumor growth inhibition. Thus, our work proposes the combination of temozolomide and roscovitine as a novel potential therapeutic possibility in glioma and other cancers associated with Cdk5 overexpression.

## Material and Methods

### Drugs and Chemicals

The drugs and inhibitors used for the current study viz., RSV and TMZ were purchased from Adooq Bioscience (USA). 3MA, Bafilomycin A1 (BaF-A1) and Chloroquine (CQ) were purchased from Sigma (Sigma Aldrich, MO, USA). Z-DEVD FMK and Z-VAD FMK were obtained from Calbiochem (Calbiochem, LaJolla, CA). 3-MA was directly dissolved in DMEM prior to use, CQ was dissolved in sterile water. Baf-A1, Z-DEVD FMK and Z-VAD FMK were dissolved in DMSO to prepare respective stock solutions.

### Cells and Culture conditions

Human GBM cell lines (U87, U373, LN18) and rat glioma cell line C6 were obtained from National Centre for Cell Science (NCCS), Pune, and maintained in cell culture facility. Other cell culture related reagents were purchased from GIBCO-life technologies (NY, USA). Cells were routinely cultured in Dulbecco’s Modified Eagles Medium (DMEM) supplemented with 10% heat- inactivated fetal bovine serum (FBS) (Gibco, NY, USA), penicillin (100 U/ml) and streptomycin (100 mg/ml) (Invitrogen Life Technologies, CA, USA) and maintained at 37 °C in a 5% CO_2_ humidified incubator (Thermo Fisher Scientific, OH, USA).

### MTT cytotoxicity assay

Cells were seeded at a density of 10,000 cells per well in 96 well plates and allowed to adhere for 24 h at 37^o^C. Following this, cells were subjected to treatment with the drugs and/or inhibitors as per the experimental requirement. Control cells were treated with vehicle (DMSO). After 72 h of drug treatment, medium was removed and 50 µl of MTT (methylthiazole tetrazolium, 1 mg/ml in 1X PBS) was added to each well and further incubated for 4 h at 37^o^C. Formazan crystals thus formed were solubilized in 50 µl iso-propanol and absorbance was measured at 570 nm using 630 nm as reference filter.

### Long term survival assay

U87 or C6 cells were plated at a density of 2000 cells/well in 6 well plate and allowed to adhere for 24 h. Further, these cells were treated with either vehicle, drug and/or inhibitors as per the experimental requirements. After 72 h, medium was removed and fresh complete medium was added. Cells were allowed to grow for 14 days with the change of media after every 3 days. Thereafter, cells were fixed with 4% paraformaldehyde for 10 min and stained with 0.05% crystal violet for 30 min at room temperature. Images were taken using digital camera (Olympus, Tokyo, Japan).

### Immunoblotting

The control and treated cells were washed with ice cold PBS thrice and lysed in ice cold cell lysis buffer (CST, MA, USA) with 1 mM PMSF. Briefly, the cells were scraped, collected and centrifuged at 3500 rpm for 5 min. Pellets were washed with ice cold PBS and lysed for 30 min on ice. The lysates were then passed through 27 gauge syringe and centrifuged at 12000 rpm for 20 min. Clear supernatants were stored at −80 °C. The protein concentration was quantified by Bradford protein assay reagent (Sigma-Aldrich, MO, USA). Equal amount of protein samples were resolved on 10–12% SDS- polyacrylamide gels and then transferred onto nitrocellulose membrane (GE Healthcare, UK). The membranes were blocked with 5% non-fat milk or 3% BSA and further probed with respective primary antibodies at 1:1000 dilution overnight at 4 °C. Blots were then washed and probed with respective HRP-conjugated secondary antibodies at a dilution of 1:2000. The blots were developed using luminescence detection reagents (Thermo Scientific, IL, USA). Whenever required the blots were stripped and reprobed. Otherwise, gels run in duplicate were probed for required proteins.

### RNA isolation, cDNA synthesis and Semi-quantitative PCR

Total cellular RNA was extracted using TRIzol reagent (Sigma-Aldrich, MO, USA) according to manufacturer’s instructions. One microgram of total RNA was converted to cDNA using cDNA synthesis kit (Takara Bio, USA) which was subsequently used for semi-quantitative PCR. The primer pairs used were as follows: Cdk5 5′-GGG AAG GCA CCT ACG GAA CTG-3′ (F), 5′-GGC GGA ACT CGG CAC ACC-3′ (R); p35 5′-ACG GTG CTG TCC CTG TCT T -3′ (F), 5′-TGG CGT TCT TGC TGT TCT GT-3′(R) and GAPDH 5′-TGA CCC CTT CAT TGA CCT CA-3′ (F), 5′-GAG ATG ATG ACC CTT TTG GCT-3′ (R). The annealing temperature used for all the primers was 58 °C.

### SiRNA transfections

Almost 80% confluent cells in 96 well plates or 35 mm plates were transfected with 100 nM or 250 nM of non specific control SiRNA or Cdk5 specific SiRNA(Santa Cruz, CA) reconstituted in SiRNA dilution buffer using Lipofectamine 2000. Six hour post transfection, medium was removed and fresh medium was added and cells were grown for 24 h and subsequently used for MTT assay or whole cell lysates were prepared for immunoblotting.

### Immunofluorescence

Glioma cells, approximately 1 × 10^4^ cells were plated on coverslips in 24 well plate and allowed to grow for 24 h. Cells were then treated with desired concentrations of TMZ for 72 h. The medium was removed from the wells and cells were washed thrice with 1X ice cold phosphate buffered saline (PBS). After that the cells were fixed with 4% PFA for 15 min at room temperature. Cells were then washed with PBS twice, followed by membrane permeabilization with ice cold acetone and methanol (1:3) for 15 min at room temperature. Cells were then washed twice with ice cold PBS followed by blocking with 3% BSA for 1 h. Cells were then incubated with primary antibodies (1:100) in blocking solution at 4 °C overnight. After that cells were washed and incubated with secondary antibody (1:400) in blocking solution for 2 h at room temperature. After washing the cells thrice with ice cold PBS, cells were incubated with DAPI for 60 sec at room temperature. The cells were then again washed with ice cold PBS and coverslips were mounted on glass slides with mounting medium and analyzed by confocal microscope (Zeiss).

### Animal experiments

All animal experiments were carried out as per the requirement and guidelines of the Committee for the Purpose of Control and Supervision of Experiments on Animals (CPCSEA), Government of India and after obtaining permission of the Institutional Animal Ethics Committee (IAEC), University of Hyderabad. Experimental Wistar rats were procured from National Institute of Nutrition (NIN), Hyderabad, India. All rats were housed in standard facilities with four rats per cage and were provided with water and food ad libitum. Rats (220–275 g) were anesthetized by intraperitoneal injection of xylazine and ketamine, 10 mg/kg and 50 mg/kg respectively. Stereotaxic implantation was performed as described previously^41^. Briefly, rats were secured in stereotactic frame and scalp incision was made. C6 glioma cell suspension (1 × 10^5^ cells in 10 µl DMEM) was injected in anesthetized rat brain (striatum region) through a drilled burr hole using a hamilton syringe and the cavity was filled with dental cement. Rats were closely monitored daily for tumor symptoms. On 7^th^ day of implantation two rats were randomly selected, sacrificed and screened to confirm the presence of tumor by histological examination using H and E staining. On 8^th^ day, the rats were randomly assigned to four groups of six rats each. Rats were treated intraperitoneally with 10 mg/Kg TMZ, 25 mg/Kg RSV or 10 mg/Kg TMZ + 25 mg/Kg RSV. Dosages of TMZ and RSV were selected based on existing literature^10,42^. Drugs were administered on alternate days in total 3 doses. Control rats were treated with vehicle (10% DMSO in saline). Rats were sacrificed on 14^th^ day of tumor implantation, perfused with ice cold PBS followed by *in situ* fixation with 4% paraformaldehyde. Brain tissues were carefully harvested and used for histology and immunohistochemical studies.

### Histology and Immunohistochemistry

Rats were sacrificed on 14^th^ day after implantation of C6 glioma cells. Brains were harvested and fixed in 4% paraformaldehyde. The brain tissues were then dehydrated in sucrose gradient of 10, 20 and 40% and sectioned in cryomicrotome (Leica CM1850). Sections of approx. 10 µm thickness were stained with haematoxylin and eosin (H&E) and 20 µm were used for IHC staining.

Immunohistochemistry was performed using IHC kit (Bio SB, CA, USA), according to manufacturers’ protocol. Briefly, pre-heated sections were hydrated thrice with PBST for 10 min each. Following this, antigen retrieval was carried out with 10 mM citrate buffer with 0.05% tween-20 (pH 6.0). Sections were incubated with 0.3% hydrogen peroxide to quench non-specific peroxidase. The non-specific binding activity was blocked by 2% BSA for 1 h at room temperature in a humid chamber. The sections were then incubated in primary antibodies (1:100) for Ki67 (CST, Danvers, USA), GFAP (CST, Danvers, USA), CD31 (CST, Danvers, USA), VEGF (Santa Cruz Biotechnology,CA) and LC3B (CST, Danvers, USA) for 72 h at 4 °C. Further, the sections were washed and incubated in Polydetector HRP label for 45 min at RT followed by incubation for 20 mins in polydetector diaminobenzidine (DAB) chromogen (Bio SB, CA, USA). Sections were then counterstained with hematoxylin and washed with double distilled H_2_O twice for 5 mins each. After subsequent dehydration in graded alcohol and xylene, sections were mounted in DPX mountant on coverslips (SRL, India). The sections were examined and the images were taken using QIMAGING camera (Micropublisher 3.3 RTV) attached to microscope (Olympus, Tokyo, Japan). Percent staining was analysed by IHC Profiler plugin in Image J software.

### Statistical analysis

Data are expressed as mean ± standard deviation. In most cases, bars represent variations within the wells of an experiment. Statistical analysis was performed using Sigma Plot 11.0 (Systat Software Inc., CA, USA). Statistical comparisons were made using Students’ two tailed-unpaired t test and p value < 0.05 was considered statistically significant.

## Supplementary information

Dataset 1

## Data Availability

All data generated or analysed during this study are included in this published article (and its Supplementary Information files).
