# Supplementary material for: Roscovitine effectively enhances antitumor activity of temozolomide in vitro and in vivo mediated by increased autophagy and Caspase-3 dependent apoptosis
Source: Sci Rep. 2019 Mar 21;9:5012. doi: 10.1038/s41598-019-41380-1 (PMC6428853; doi:10.1038/s41598-019-41380-1)
Supplement: Supplementary file 1 — Dataset 1 [file 41598_2019_41380_MOESM1_ESM.pdf]

Roscovitine effectively enhances antitumor activity of temozolomide in vitro and in vivo mediated by increased autophagy and Caspase-3 dependent apoptosis.

Vimal Pandey*, Nikhil Ranjan*, Parimala Narne, Prakash Babu Phanithi#

Laboratory of Neuroscience, Department of Biotechnology & Bioinformatics, School of Life Sciences, University of Hyderabad, Hyderabad, AP, India

# Corresponding Author

Phone: 0091-40-23134584; 23013336; Fax: 0091-40-23010120

Email: prakash@uohyd.ac.in; prakashbabuphanithi@gmail.com

*Equal contribution

Running title: Roscovitine enhances temozolomide chemo-sensitivity in glioma

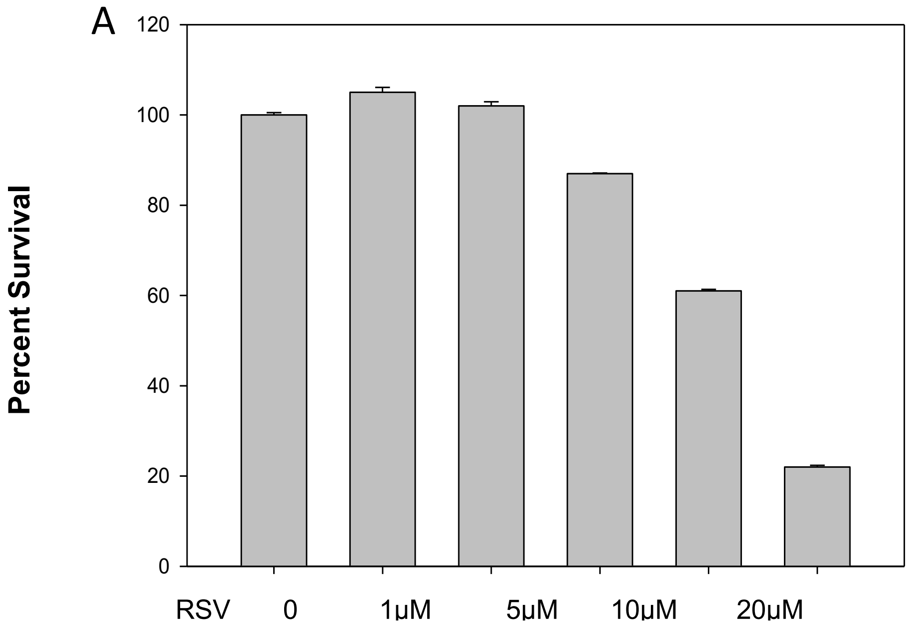
A
Percent Survival
120
100
80
60
40
20
0
RSV
0
1µM
5µM
10µM
20µM

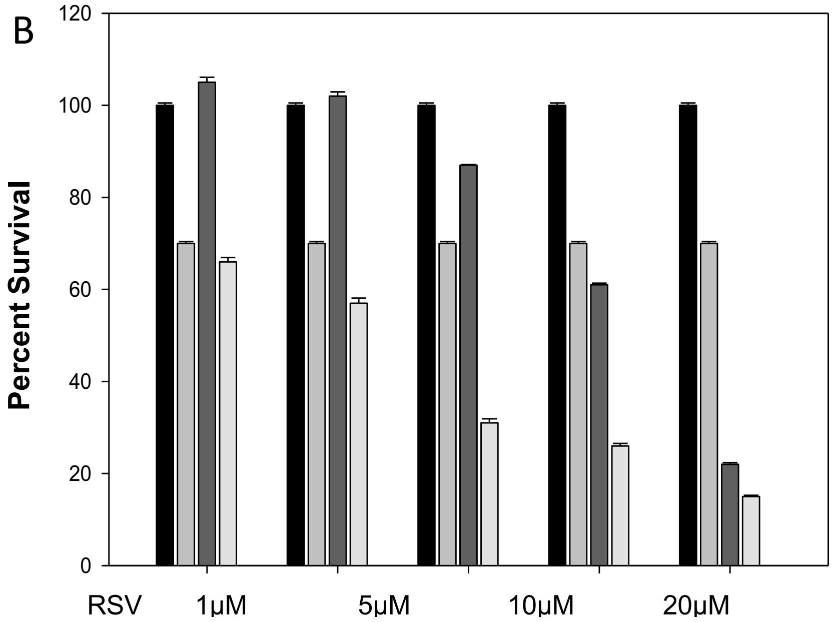
B
Percent Survival
120
100
80
60
40
20
0
RSV
1µM
5µM
10µM
20µM

C

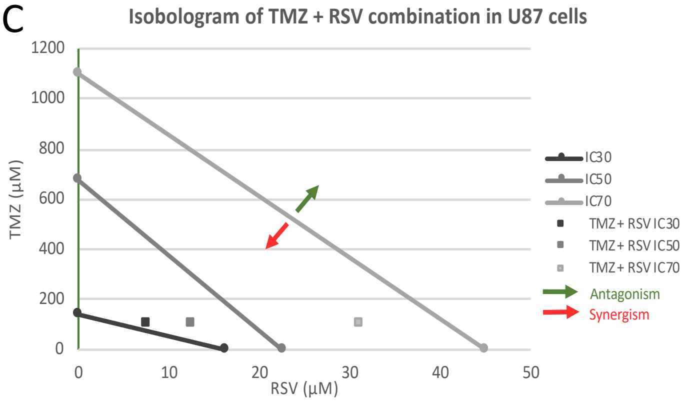

Combination I= TMZ (100mM)+RSV			
IC	TMZ	RSV	CI
30	100	7.5	1.7
50	100	12.55	0.7
70	100	31	0.7

Supplementary Figure 1: Roscovitine reduces glioma cell viability and potentiates the cytotoxic effects of TMZ on glioma cells. A) Effect of RSV on cell viability in LN 18 human glioma cell lines. The cells were treated with increasing concentrations of RSV for 72 h and cell viability was assessed by MTT assay. B) Effect of TMZ and RSV co treatment on cell viability of LN18 glioma cell lines. Cells were treated first with indicated concentrations of RSV followed by co-treatment with 100 µM TMZ. After 72h incubation with the drugs cell viability was measured by MTT assay. C) Isobologram and combination index (CI) of TMZ and RSV in U87 cells.The CI was calculated using the formula:

$$CI = \frac{C_{A,X}}{IC_{X,A}} + \frac{C_{B,X}}{IC_{X,B}}$$

where CA,X and CB,X are the concentration of TMZ(A) and RSV(B) used in combination to achieve x % drug effect. ICX,A and ICX,B are the concentrations for single agents to achieve the same effect. A CI of less then, equal to, or more than 1 indicates synergic, additive or antagonistic effect, respectively.

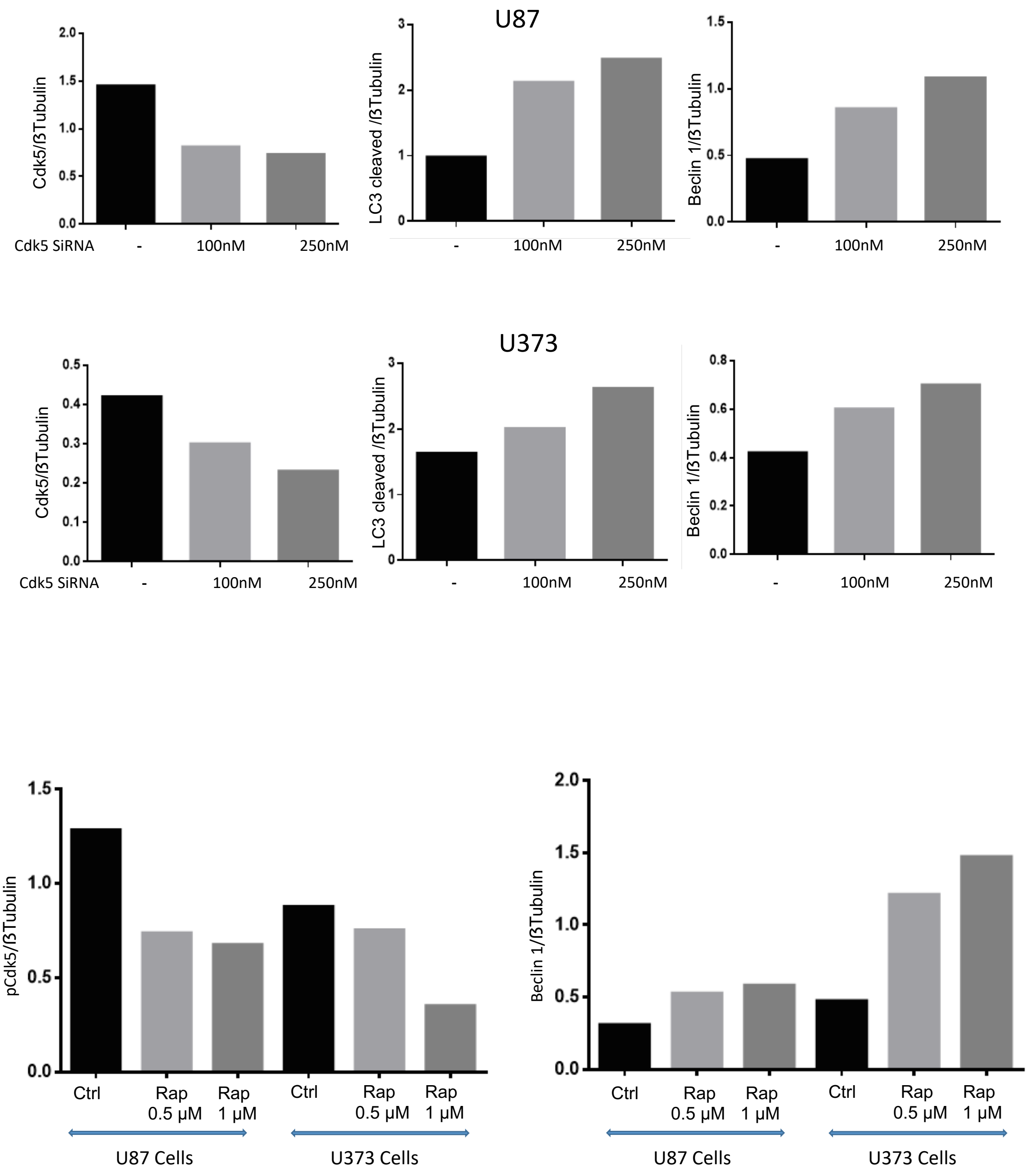

Supplementary Figure 2 : Densitometric analysis of immunoblots from Figure 4. The intensities of individual protein bands were quantified using ImageJ software, were normalized to the intensities of their respective loading controls and were then plotted as histograms.

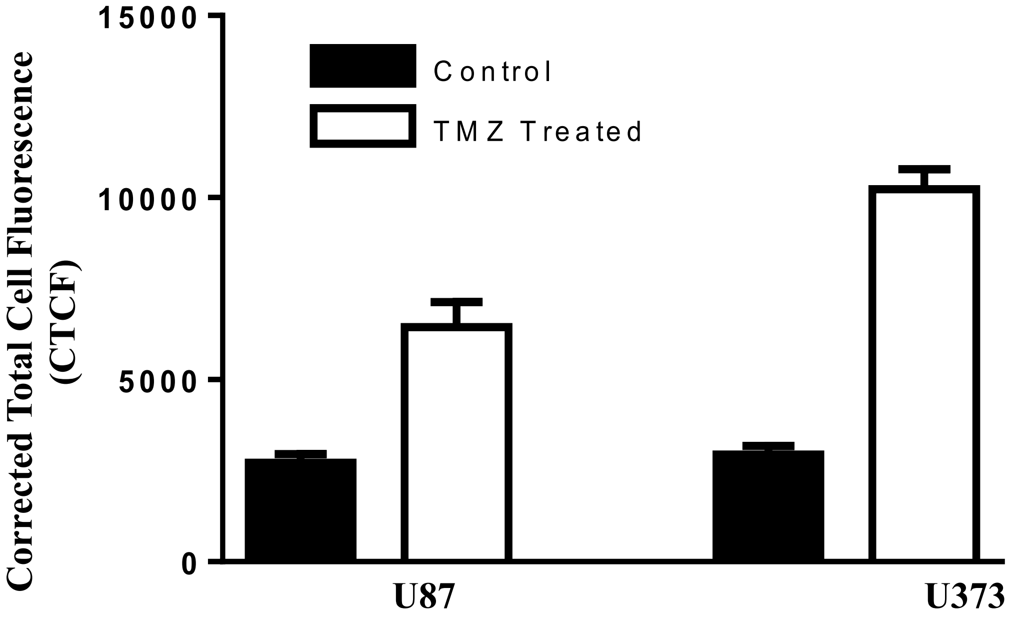

Supplementary Figure 3: Immuno-fluorescent staining quantification data for LC3B in U87 and U373 untreated and TMZ treated cells. (Fig. 6)

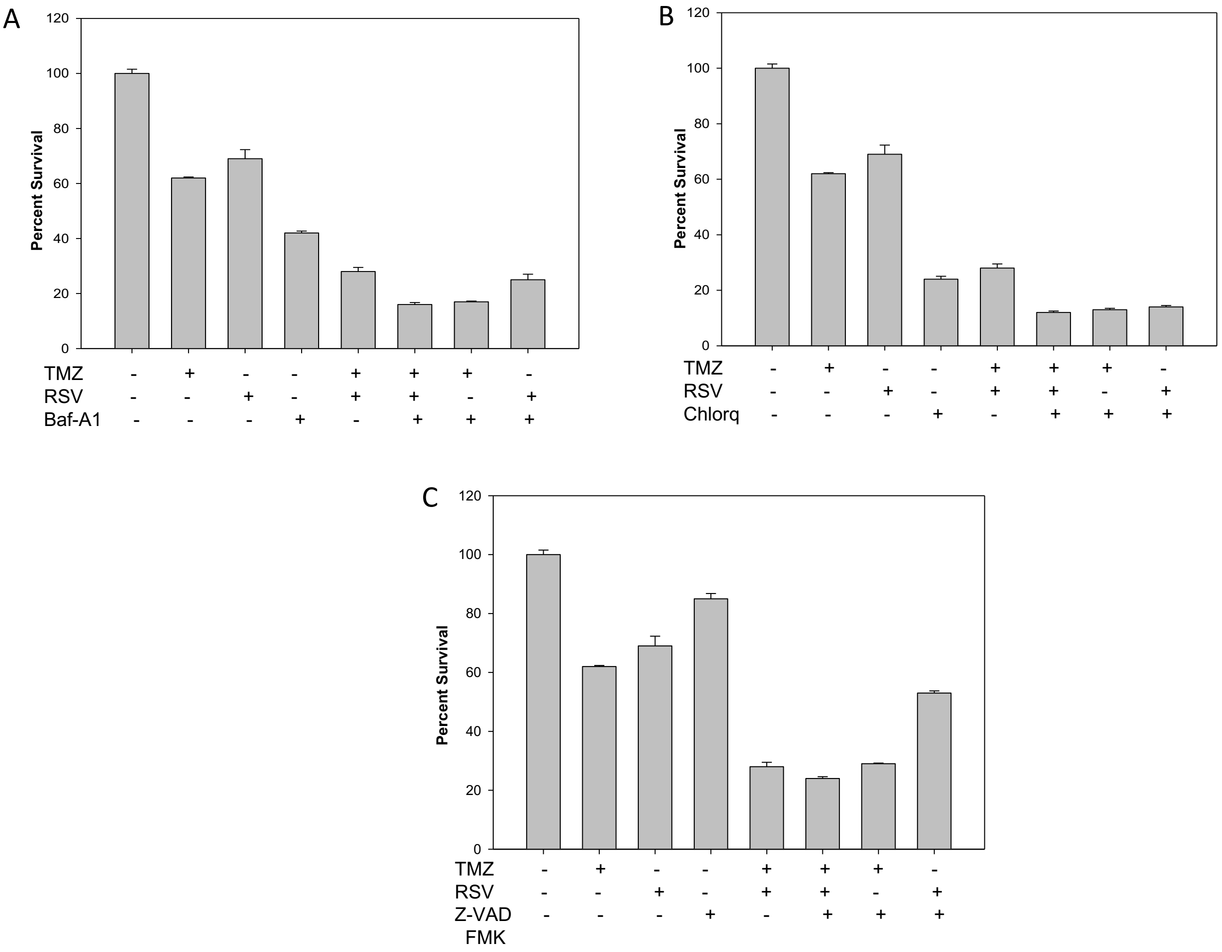

Supplementary Figure 4: Effect of A) Bafilomycin , (B) Chloroquine, autophagy inhibitors and (C) Z-VAD FMK, a pan caspase inhibitor on the cytotoxicity of various drug treatments in C6 cells. For this assay, C6 cells were pretreated with indicated concentrations of inhibitors for 2 h and then treated with TMZ, RSV or TMZ+RSV for 72h. This was followed MTT assay to determine percent cell survival.

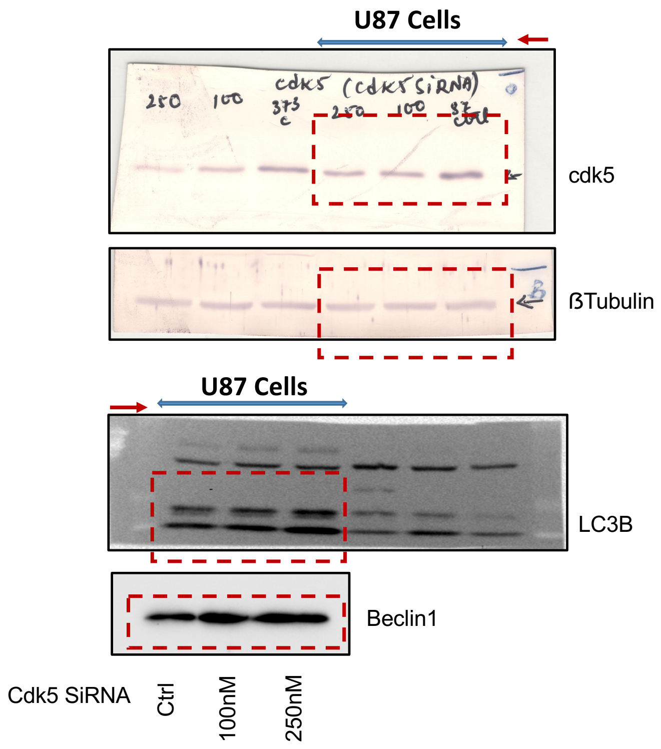

Supplementary Figure 5: Full Length blots of Cdk5, ß tubulin, LC3B and Beclin 1 (Fig. 4, U87 cells)

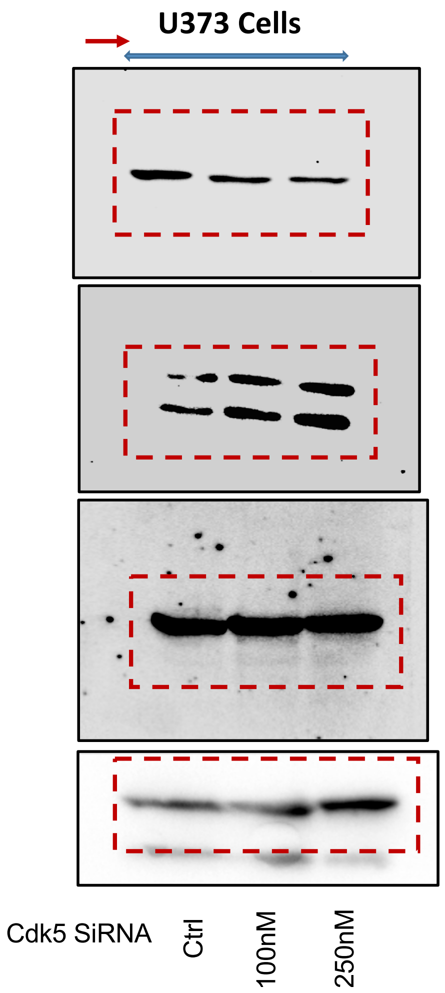

Supplementary Figure 6: Full Length blots of pCdk5, ß tubulin and LC3B (Fig. 4, U373 cells)

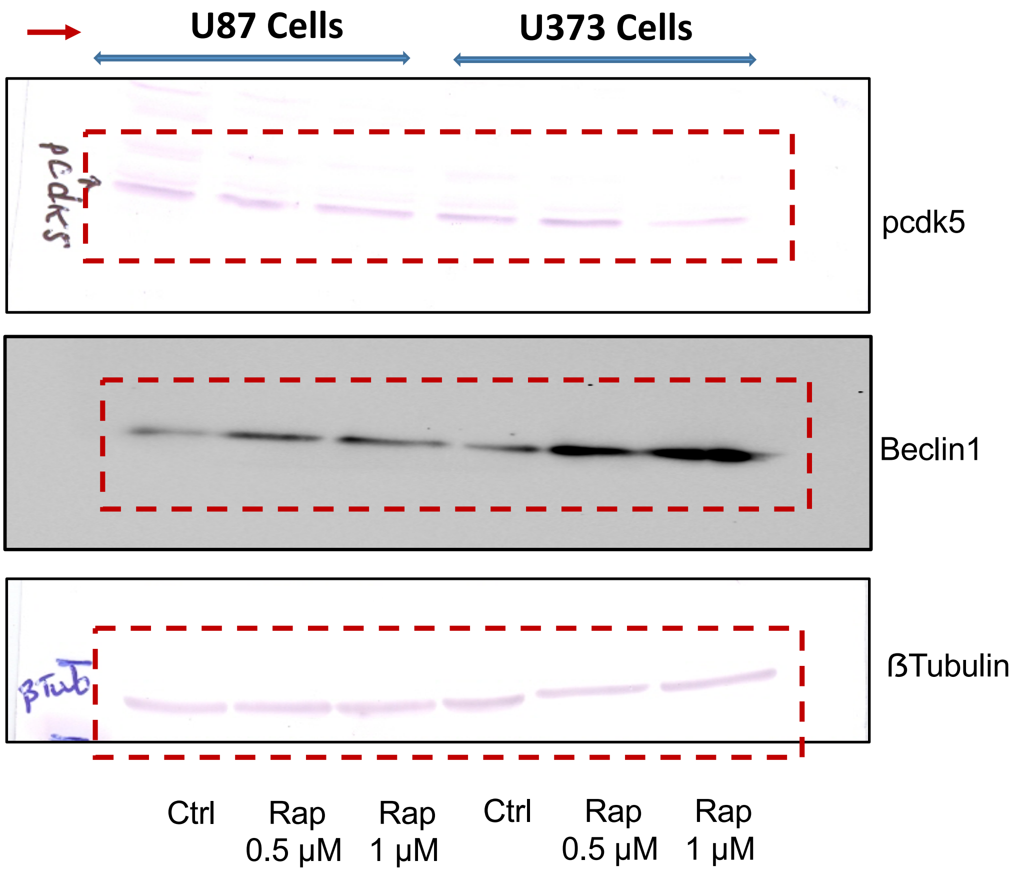

Supplementary Figure 7: Full Length blots of pCdk5, Beclin 1 and ß tubulin (Fig. 4)

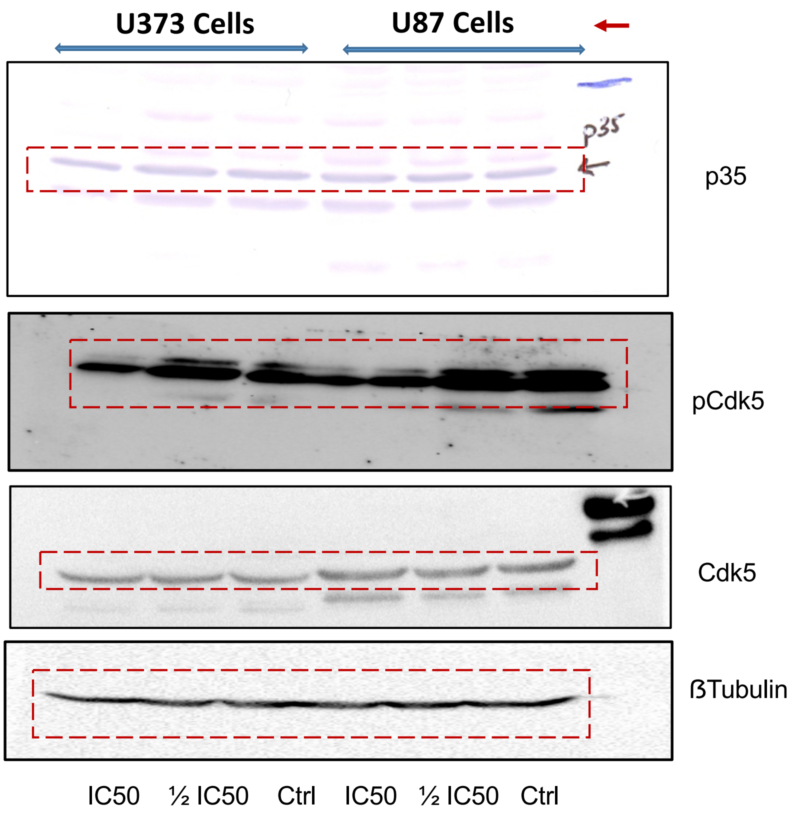

Supplementary Figure 8: Full Length blots of p35, pCdk5, Cdk5 and ß tubulin (Fig. 5)

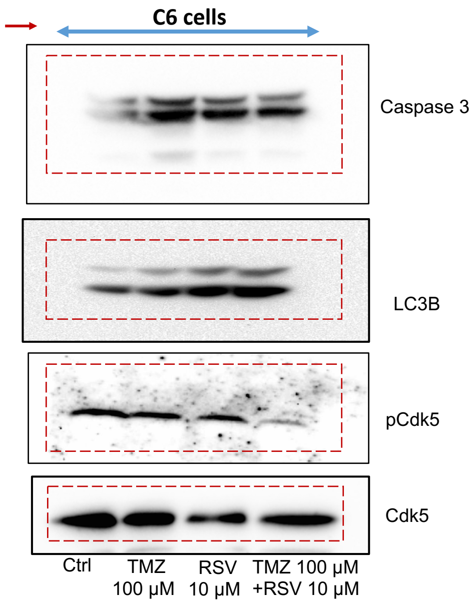

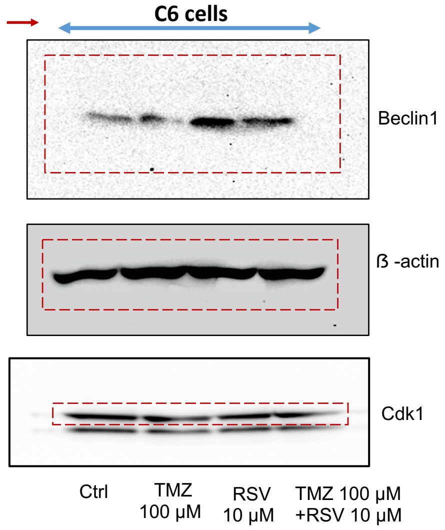

Supplementary Figure 9: Full Length blots of Caspase 3, LC3B, pCdk5, Beclin1 and ß tubulin (Fig. 5)

IHC Quantification Data (IHC profiler Plugin, Image J software)

Supplementary Table 1: Ki67

	High Positive	Positive	Low Positive	Negative	Staining Result
Ctrl	52.41 ± 2.596	45.72 ± 3.330	1.867 ± 0.7337	0.0 ± 0.0	High Positive
TMZ	11.57 ± 1.367	25.40 ± 4.360	33.95 ± 4.375	24.09 ± 2.369	Positive
RSV	2.217 ± 0.8296	12.48 ± 2.127	44.70 ± 4.934	40.61 ± 1.977	Low Positive
TMZ+RSV	2.664 ± 1.570	7.150 ± 0.8564	17.63 ± 0.04875	72.56 ± 2.476	Negative

Supplementary Table 2: GFAP

	**High Positive**	**Positive**	**Low Positive**	**Negative**	**Staining Result**
Control	50.89 ± 3.949	35.58 ± 3.198	10.73 ± 0.3554	2.805 ± 0.3957	High Positive
TMZ	4.796 ± 0.1391	8.057 ± 1.546	29.73 ± 4.665	57.42 ± 6.350	Low Positive
RSV	1.514 ± 0.6465	8.156 ± 1.869	34.96 ± 4.570	55.37 ± 3.348	Low Positive
TMZ+RSV	0.0001788 ± 0.0001358	0.8645 ± 0.05705	13.80 ± 0.3909	85.34 ± 0.4478	Negative

Supplementary Table 3: CD31

	**High Positive**	**Positive**	**Low Positive**	**Negative**	**Staining Result**
Ctrl	16.07 ± 1.061	34.86 ± 6.889	38.20 ± 7.599	10.87 ± 0.3510	Positive
TMZ	0.07045 ± 0.02108	5.377 ± 0.9341	37.01 ± 2.751	57.54 ± 3.664	Low Positive
RSV	0.002605 ± 0.002489	1.100 ± 0.1347	30.17 ± 2.884	68.73 ± 3.021	Low Positive
TMZ+RSV	3.743 ± 0.7390	8.541 ± 1.327	43.05 ± 6.636	44.67 ± 4.570	Low Positive

Supplementary Table 4: VEGF

	High Positive	Positive	Low Positive	Negative	Staining Result
Ctrl	11.30 ± 0.8798	35.19 ± 0.9436	49.76 ± 1.171	3.738 ± 0.6522	Positive
TMZ	7.798 ± 1.298	26.41 ± 4.372	37.10 ± 1.784	28.69 ± 3.885	Low Positive
RSV	6.860 ± 2.471	24.44 ± 2.907	44.88 ± 4.356	23.83 ± 1.022	Low Positive

TMZ+RSV	1.867 ± 0.9182	10.02 ± 0.6541	49.37 ± 2.845	38.74 ± 1.273	Low Positive

Supplementary Table 5: LC3B

	High Positive	Positive	Low Positive	Negative	Staining Result
Ctrl	6.716 ± 2.642	10.19 ± 1.166	38.85 ± 3.688	44.25 ± 0.1209	Low Positive
TMZ	16.03 ± 2.629	16.58 ± 2.085	42.88 ± 0.4332	24.51 ± 0.1106	Positive
RSV	12.66 ± 1.415	31.29 ± 3.071	35.29 ± 1.704	20.77 ± 2.783	Positive
TMZ+RSV	21.14 ± 2.994	36.04 ± 5.253	27.72 ± 3.495	15.10 ± 4.752	High Positive
